# Declarative Memory Impairment and Emotional Bias in Recurrent Depression with a Seasonal Pattern: The Interplay between Emotion and Cognition in Seasonal Affective Disorder

**DOI:** 10.3390/brainsci12101352

**Published:** 2022-10-05

**Authors:** Carla Iorio, Francesca Pacitti, Alessandro Rossi, Paola Iorio, Assunta Pompili

**Affiliations:** Department of Biotechnological and Applied Clinical Sciences, University of L’Aquila, 67100 L’Aquila, Italy

**Keywords:** seasonal affective disorder, declarative memory, depression, emotion, cognition, seasonality

## Abstract

Seasonal Affective Disorder (SAD) is a subtype of Major Depressive Disorder (MDD) with a seasonal pattern. Although it is a pathological condition limited to specific seasons of the year, during the symptomatic period, patients may experience a significant impairment of well-being and daily quality of life as a result of the depressed mood, associated with other symptoms defined as atypical of MDD. While extensive evidence of memory deficits has been found in MDD, explicit memory impairments in SAD are insufficiently studied. This study aims to investigate the cognitive processing of emotional stimuli in women with SAD, in particular the interplay between emotions and declarative memory. One hundred and twenty young women, screened from an initial number of 1125 university students, were divided into two groups, an experimental one that included 60 medically untreated women affected by “winter type SAD” and a control group of 60 non-SAD women. Different subjects were randomly submitted to two types of audio–visual stories, neutral or arousal, and then their memory performances were analyzed by means of a free-recall test and a recognition memory test. In both the free-recall test (*p* < 0.008) and in the recognition memory test (*p* < 0.002), the SAD group showed impaired memory performances. Taken together, our novel key findings suggest that SAD is characterized by impairment in declarative memory and attentional bias for emotional negative stimuli.

## 1. Introduction

In some people, seasonal changes can bring on mood and behavioral symptoms that can negatively influence their ability to function [1], and the individual tendency towards seasonal variations, the so-called seasonality, is evident in various medical conditions [2,3,4,5,6,7,8].

For decades, it was assumed that there was a relationship between the onset of symptoms of depression and the seasonal variations, but it was only in 1984 that Rosenthal et al. [9] formulated the diagnostic criteria for Seasonal affective disorder (SAD). In the most recent edition of the Diagnostic manual of Mental Disorders (DSM-5, American Psychiatric Association, 2013) [10], SAD is identified as a subtype of Major Depressive Disorder (MDD) characterized by the recurrence of depressive episodes with a seasonal pattern for at least two years, resulting in changes in mood tone and behavior. Its onset and termination occur in approximately the same period each year and although it shares many characteristics with MDD, such as depressed mood or lack of pleasure and interest, SAD shows many differences in a number of symptoms defined as atypical, such as hyperphagia, weight gain, and hypersomnia.

The prevalent form of SAD, the so-called “winter SAD”, consists of clinical manifestations that appear in late fall, reach their maximum expression in the winter, and resolve or improve during the summer season. The intensity of symptoms is variable, and Kasper et al. (1989) [1] proposed a continuum in seasonality, with the symptom patterns varying from very significant to subclinical (subsyndromal SAD).

Epidemiological studies support the hypothesis that individuals residing at higher latitudes have a greater risk of developing the winter form of SAD. Its prevalence ranges from 1.4% to 9.7% in North America and from 1.3% to 3.0% in Europe depending on latitude [11].

The etiology of this condition includes both biological/genetic and psychological hypotheses. The biological and genetic theories underlying SAD are focused on the relationship between the daylight duration, shorter in fall and winter, and the SAD symptoms onset, and indeed light therapy seems to be effective in the treatment of some SAD individuals [12]. The light–dark cycle is the main environmental cue that synchronizes daily biological rhythms, and the change of season can induce abnormalities, altering melatonin levels and leading to an alteration of sleep and mood. According to the phase-shift hypothesis, the rhythm abnormalities in SAD are determined by the nocturnal production of melatonin by the pineal gland, with a phase shift in autumn/winter, related to the different light–dark periods compared to spring/summer [13,14,15].

The genetic basis involves the circadian clock genes that regulate the suprachiasmatic nucleus [16], and the gene that regulates retinal photo pigments, such as melanopsin, which is found in retinal cells that project onto the central circadian clock, a factor that contributes to the decreased sensitivity to light in SAD [17]. Many studies have evidenced a deficit in serotonin transmission, which may be reversed by exposure to bright light [18,19,20,21].

Although most studies attempting to explain SAD are focused on biological and genetic factors, some research indicates that psychological factors also play an important role in the etiology of this disorder. Attentional bias, rumination, and automatic thoughts may contribute to the psychological vulnerability of SAD subjects [22,23,24,25].

A great deal of experimental evidence suggests that in nonseasonal depression, patients suffer cognitive symptoms in numerous domains, such as processing speed, attention, executive function, and memory [26,27]. On the contrary, cognitive problems in seasonal depression have been insufficiently studied. The limited research conducted on cognitive deficits in SAD has not been entirely consistent and has often involved a small number of subjects. Existing data in the scientific literature highlight in some cases a lack of and in others the presence of clinically significant cognitive deficits in SAD compared to healthy controls [28,29,30,31,32,33].

In one of the most significant studies, Sullivan and Payne (2007) [34] found that both SAD and MDD were associated with cognitive deficits. In fact, both disorders were associated with reduced cognitive performance and difficulties that individuals experience in typical everyday situations (e.g., forgetting names or misinterpreting directions) that are related to deficits in controlled processes, such as attention and working memory. These findings highlight that individuals with SAD may exhibit cognitive impairments similar to those that characterize nonseasonal depression.

Our study aimed to analyze the affective and cognitive components in memory processes in a medically untreated sample of young women with SAD. The choice to select only untreated participants allowed us to avoid cases with attenuation or regression of the symptoms due to pharmacological or psychotherapeutic treatments. We used the Seasonal Pattern Assessment Questionnaire (SPAQ) to diagnose SAD, while we ruled out other concomitant psychiatric conditions with a screening interview conducted by a psychiatrist or a psychotherapist and used the Beck Depression Inventory-II (BDI-II).

The scientific literature lacks research on the effects of SAD on long-term memory, while little previous research highlighted short-term memory impairments in SAD [28,30,34].

In particular, we aimed to analyze the interplay between emotions and declarative memory. It is well known that emotional content can affect attention and therefore memory, interacting with the processes of encoding and consolidation of information. Traditionally, emotion affects declarative memory according to two orthogonal dimensions, arousal, and valence. In particular, emotional arousal has an immediate role in encoding, and this probably reflects the influence of attentional processes on memory [35]. Stress, anxiety, and other emotions can profoundly influence key elements of cognition, such as selective attention, working memory, and executive control. In turn, cognitive circuits involved in attention, working memory, and executive functions can contribute to emotional regulation: cognitive systems regulate emotions, and emotional systems regulate cognition. The influence of emotions on memory has been extensively demonstrated using various types of material, which include emotional stories [36,37], emotional and neutral images [38,39], and emotional facial expressions [40]. These types of materials can be easily manipulated in a research laboratory, allowing for controlled and easily reproducible exposure, both in terms of timing and intensity.

More specifically, we examined the effects of emotional stimuli on declarative memory in young women with SAD, using two types of stories (neutral and arousal) administered in audio–visual mode. The stories [41] were previously adapted by our research group to an Italian sample [37], and both versions were accompanied by the same set of slides but with two different narratives. In addition to images, narrative recall represents a very useful measure of memory and recognition. Based on previous studies in different contexts, in which the authors have used these stories [36,42,43], we predicted that the control group would exhibit enhanced memory for the details of the arousal story (AS), containing negative arousal stimuli, compared to the neutral story (NS). In fact, the arousal of negative stimuli of the AS would facilitate the encoding and consolidation of memory, compared to neutral information. Both the AS and the NS can allow analysis of declarative memory in the SAD group, as well as the role of emotional stimuli, and we hypothesized that (1) participants with SAD would exhibit memory impairments, with worse performance in memory tasks, compared to the control group and (2) they could show an attentional bias for negative stimuli of the AS compared to the controls.

## 2. Materials and Methods

### 2.1. Participants

One thousand one hundred and twenty-five women were involved in this study, as SAD is more likely to occur in women than in men [11]. They were all students from the University of L’Aquila, and the research project was approved by the Internal Review Board of the University of L’Aquila. The participants received course credit for their contribution to the study. All subjects were initially screened through a standardized interview to check for any health problems or drug use. Exclusion criteria included any major neurological or psychiatric illness and substance abuse. They were also invited to fill out two self-administered questionnaires, the SPAQ, and the BDI-II.

The SPAQ is a retrospective self-administered questionnaire [9] designed to identify individuals with a particular tendency toward seasonal sensitivity. Subjects were asked to rate seasonal variation in six areas—sleep, social activity, mood, weight gain, eating habits, and energy level—on a 5-point Likert scale ranging from 0, no changes, to 4, extremely marked changes. The scores were used to calculate a global seasonality score (GSS), which can range from 0 to 24. The cutoff was set at 11 points, and the presence of SAD also required that participants experienced the seasonal change as a problem of at least a moderate degree of 2 points, on a scale ranging from 0, no problem, to 5, disabling [1]. In addition, the questionnaire allowed for the identification of the type of SAD, that is, the winter or summer type. The GSS has been shown to have acceptable reliability and validity in epidemiological studies [44,45,46].

The BDI-II [47] is a questionnaire originally designed by Beck et al. (1961) [48] to evaluate the presence and degree of depressive symptomatology in adolescents and adults, and it has been used to measure nonseasonal depression. It is composed of 21 items, with a 4-point scale indicating the degree of severity (0 = not at all; 3 = extreme form of each symptom). The scores range from 0 to 36, and the cutoff is set at 16 points [47]. We used the BDI-II to exclude depressed subjects to be sure that our results are about participants with SAD and not MDD.

Based on the screening interview and the questionnaire results, we selected 120 female subjects with the “winter type of SAD”, dividing them into two groups: 60 with SAD (GSS ≥ 11 and BDI-II < 16) and 60 as a control group (BDI-II < 16, no seasonal symptoms). Only subjects residing in central and southern Italy were considered to reduce the geographical variability between the participants, as it is well known that SAD varies with the variation of the earth’s latitude. 

As stated in the introduction, all subjects were medically untreated. Seven subjects with high SPAQ scores were excluded. Six of them reached a high score in the BDI-II and were under pharmacological treatment, while one was affected by summer-type SAD. As confirmed in our study, the winter type is by far the most frequent form of SAD, and we focused our attention on participants with winter-type SAD, therefore excluding the one subject with summer SAD.

The participants were screened during the period between January 2021 and October 2021. The selected subjects were then tested between November 2021 and January 2022, as the symptomatology related to SAD should manifest in the autumn/winter period.

All participants read and signed informed consent before taking part in this research, having an adequate understanding of the study aims and the experimental procedures. All the data were grouped and analyzed anonymously.

### 2.2. Stimulus Material

**Figure 1:**
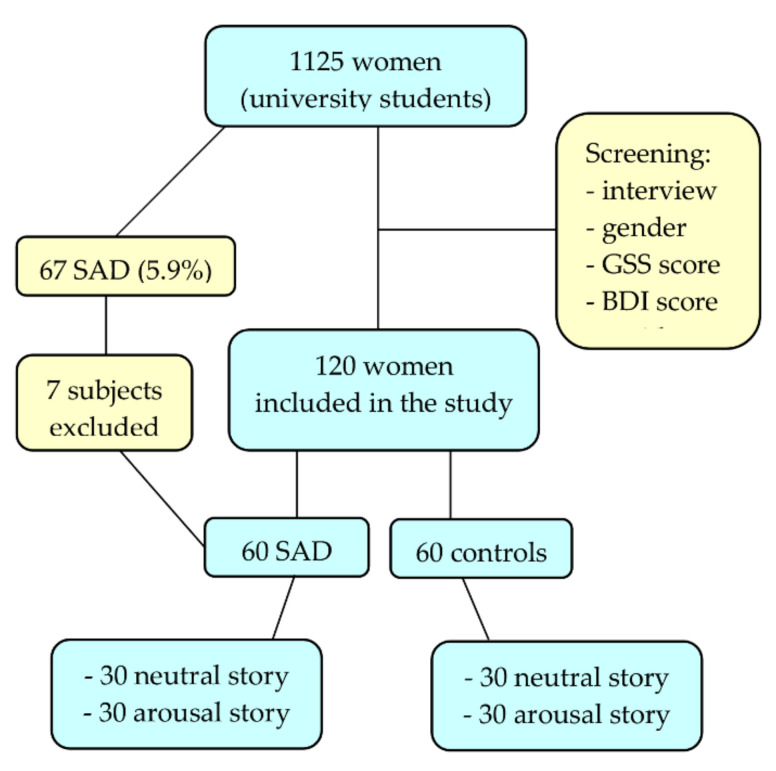
Sampling flow chart.

The stimulus material consisted of an adaptation of an instrument previously used [37,41,43], a brief story accompanied by a narrative with two different versions, one neutral and one arousal. The sixty subjects of each group, SAD and control, were randomly divided into two subgroups (*n* = 30); therefore, there were four final groups: SAD-AS, SAD-NS, control-AS, and control-NS. Each member of a specific group viewed and listened only to the AS or the NS version of the story (Figure 1).

The story comprised 11 images, identical in the two versions, which can be divided into 3 phases. Phase 1 (the first four slides, 1–4) and phase 3 (last three slides, 9–11) consisted of relatively nonemotional material and were identical in their narratives, while the narratives of phase 2 (middle four slides, 5–8) were very different in the two versions. A professional male actor narrated both versions in a flat, unemotional voice, and the slides had been previously adapted to the Italian environment [37]. 

The stories were about a mother and her young son, who were going to visit the boy’s father at the hospital where he works. In the neutral version, on their way, they see a car accident that caught the attention of the child. When the child arrives at the hospital, he is invited to attend an exercise conducted by the doctors on a child with fake leg injuries, carried out by a specialized make-up artist. In the arousal version, the boy himself is the victim of a serious car accident in which he is critically injured and operated on at the hospital. 

Phase 2 included two images (slides 7 and 8) of the International Affective Picture System (IAPS) [49] representing the surgeon in the operating room and the severely injured legs of the child. In the AS, the two images have a negative valence with high arousal because they are associated with a negative narrative, while they have a neutral valence in the NS. 

The slides were projected utilizing a multimedia system consisting of a notebook with a color screen SVGA full HD (1920 × 1080), an Epson EB-1945W projector, and a Logitech Z623 THX 2.1 speaker system with a subwoofer. The images were sequenced using Microsoft PowerPoint 2016 (Office 2016), integrating video signals with audio signals previously recorded in digital format.

### 2.3. Procedure

Before the experimental session, all participants had an interview with the psychiatrist and filled the BDI-II again to check for changes in the subject’s status.

All 120 subjects read and signed an informed consent form to participate in this research and were randomly assigned to the arousal or neutral condition. In both the arousal and the neutral situations, they were instructed to pay attention to each slide of the story and to the narration accompanying it for the duration of the presentation as if they were watching a movie.

#### 2.3.1. Emotional Rating

At the end of the stimulation sequence, subjects were requested to judge their reaction to the story previously seen. The emotional rating, given on paper, was from 0 (“not emotional”) to 10 (“highly emotional”), and they were asked to mark the scale at the appropriate point. They were then invited to return after one week for a surprise free-recall test for story elements, without any indication that their memory would be tested to avoid performance influences due to voluntary material consolidation. They were also required not to discuss the experiment with anyone in order not to compromise the results.

#### 2.3.2. Memory Test

The memory tests consisted of a free-recall test and a recognition memory test.

##### Free-Recall Test

Just before starting the free-recall test, participants were informed that what they remembered about the story seen a week earlier would be analyzed. They were asked to recall as many details as possible, and each participant’s narrative was registered anonymously using a tape recorder. According to the criteria used to evaluate the memory of the stories [37], the narration accompanying each slide was divided into segments and the records were analyzed using a scoring template including the contents of each slide for both the neutral and arousal versions. For example, the phrase of the first slide “A mother and a child are leaving their home in the morning” was divided into three key segments: (1) A mother and a child, (2) are leaving their home, (3) in the morning. The total score for each slide and the total free recall were obtained by adding the scores attributed to each segment remembered. Only unambiguous descriptions were counted.

##### Recognition Memory Test

In addition to the free-recall test, all participants were subjected to a recognition test using a questionnaire on the story consisting of 4–8 questions about each slide, with a total of 65 multiple-choice questions, and the attribution of one point for each correct answer (score from 0 to 65). Again, the purpose was to analyze what was remembered of the many details that appeared during the projection of the slides through specific questions about the scenes seen.

#### 2.3.3. Statistical Analysis

All data were analyzed using IBM SPSS Statistics for Windows software (version 25.0, IBM Corp, Armonk, NY, USA). We first verified the normal distribution of the dataset using the Kolmogorov–Smirnov test to apply the correct statistical tests. All data were normally distributed and therefore, we used analysis of variance (ANOVA) to determine whether there was an interaction effect between the two independent variables (groups, SAD and controls, and story type, AS and NS), and the dependent variable (memory scores).

The sociodemographic characteristics of the sample and the GSS and BDI-II scores were assessed using one-way ANOVA.

The emotional rating scores, the total results of the free recall test, and the recognition memory test were analyzed using two-way ANOVA, with the groups (SAD, Control) and the stories (AS, NS) as independent factors, and the interaction effects were assessed. 

In order to assess differences in story phases, significant results were then analyzed using two-way ANOVA, with the group (SAD-arousal, control-arousal; SAD-neutral, control-neutral) as a between-subject factor and the three different phases of the story (phase 1, 2, and 3) as a within-subject factor.

Tukey’s Honestly Significant Difference (HSD) post-hoc test was applied to determine significance. 

We investigated the correlation between the GSS scores and the free-recall and questionnaire scores using Pearson’s coefficient correlation.

A binary logistic regression was used to examine whether age, residence, GSS, and BDI were associated with the likelihood to have an SAD diagnosis (results are shown in supplementary material).

Results are presented as means ± standard error mean (SEM) or standard deviation (SD). The level of significance was set at 5% (*p* < 0.05).

## 3. Results

### 3.1. Demographic and Clinical Characteristics of the Sample

A comprehensive overview of the demographic variables and of the questionnaire scores (age, number of years of residence in central and southern Italy, GSS, and BDI-II scores) is presented in Table 1.

We found no significant differences between the four groups with respect to either age (mean age: 22.50 ± 2.676; F_3,116_ = 15.376, p = 0.091) or the number of years of residence in central and southern Italy (18.19 ± 5.204 years; F_3,116_ = 0.097, *p* = 0.961). The SAD group presented a GSS score significantly higher than the control group (mean score of SAD: 14.60 ± 2.726; mean score of control: 8.50 ± 3.735. F_1,118_ = 104.426, *p* < 0.001). In the SAD group, a BDI score significantly higher than that of the control group was also evidenced (mean score of SAD: 8.80 ± 3.545; mean score of control: 5.90 ± 3.251. F_1,118_ = 21.811, *p* < 0.001).

### 3.2. Emotional Rating of the Stories

An overall two-way ANOVA with group (SAD, control) and the story type (AS, NS) as independent factors evidenced a main effect of the story type on the emotional rating (F_1,116_ = 117.744; *p* < 0.0001, η^2^ = 0.504). 

There was a significant interaction of group x story type (F_1,116_ = 10.185; *p* < 0.002, η^2^ = 0.81), and the subjects with SAD judged the AS to be more emotional than the control group (F_1,58_ = 6.684; *p* < 0.01).

**Figure 2:**
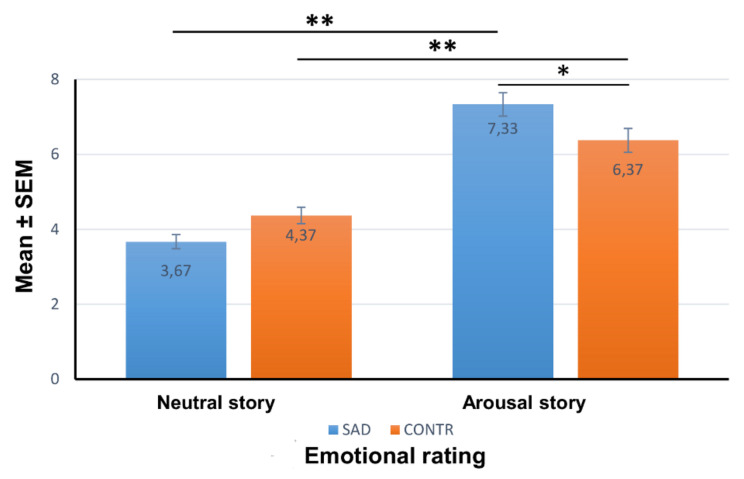
Emotional rating. Mean (±SEM) ratings of the emotional reaction to the neutral and arousal stories in the SAD and control groups. * *p* < 0.01; ** *p* < 0.001.

We next explored the emotional rating in the different groups. As we expected, the results showed that there was a significant difference between the AS and the NS ratings. In fact, both the SAD group (F_1,58_ = 184.684; *p* < 0.001) and the control subjects (F_1,58_ = 20.008; *p* < 0.001) judged the AS to be more emotional than the NS. There was no significant difference between the two different groups regarding the emotional rating of the NS (F_1,58_ = 3.687; *p* = 0.090, n.s.). Results are reported in Figure 2.

### 3.3. Free Recall of the Stories

#### 3.3.1. Total Free Recall

An overall two-way ANOVA with group (SAD, control) and story type (AS, NS) as independent factors was first conducted to analyze the total recall of the slides from the arousal and the neutral version of the story and then to analyze the slide recall in the different groups separately. 

The total recall of the slides showed a main effect of story type on memory scores (F_1,116_ = 33.260, *p* < 0.0001; η^2^ = 0.223), indicating that participants recalled more slides from the AS compared with the NS. There was also a significant group effect on memory scores (F_1,116_ = 9.397, *p* < 0.003; η^2^ = 0.075), as the control group recalled a significantly higher total number of slides than the SAD group (F_1,118_ = 7.302; *p* < 0.008). There was no interaction of group x story type (F_1,116_ = 2.594, p = 0.110; η^2^ = 0.011).

**Figure 3:**
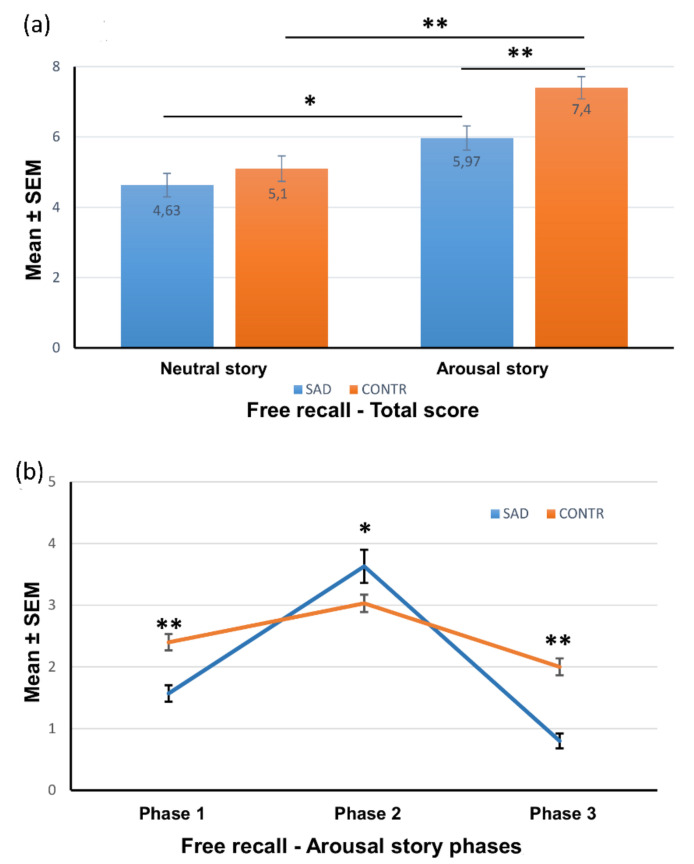
Free recall scores. (**a**) Score (mean ± SEM) of the total story recall of the participants under neutral and arousal conditions. * *p* < 0.01; ** *p* < 0.001. (**b**) Recall scores (mean ± SEM) for each phase of the story, in subjects assigned to the arousal condition. * *p* < 0.05; ** *p* < 0.001.

We next explored the total slide recall in the two groups separately. As we expected, the results evidenced that both the SAD (F_1,58_ = 7.736; *p* < 0.01) and the control group (F_1,58_ = 31.608; *p* < 0.001) remembered a significantly higher number of slides of the AS compared with the NS. There were no significant differences between the two groups for the NS (F_1,58_ = 0.895; *p* = 0.348, n.s.), although the SAD group remembered a smaller number of slides than the controls. There was a significant difference for AS, with the SAD group remembering a significantly lower number of slides in comparison with the controls (F_1,58_ = 13.368; *p* < 0.001) (Figure 3a). 

#### 3.3.2. Free Recall for Each Phase

We next examined the recall of slides in each phase of the stories using two-way ANOVA with group (SAD-AS, control-AS; SAD-NS, control-NS) as a between-subject factor and the story phase (1, 2, 3) as a within-subject factor. 

The overall ANOVA for all three phases showed a main effect of story phase on memory scores (F_2,232_ = 63.998; *p* < 0.001, η^2^ = 0.356) and a significant group x story phase interaction (F_6,232_ = 19.182; *p* < 0.001, η^2^ = 0.332).

A series of separate ANOVAs evidenced that subjects with SAD remembered a significantly lower number of slides in the neutral phases, 1 and 3, compared with the control group (F_1,58_ = 19.766; *p* < 0.001 and F_1,58_ = 42.041; *p* < 0.001, respectively) but remembered a significantly higher number of slides from the emotional phase 2 (F_1,58_ = 3.618; *p* < 0.05) (Figure 3b).

### 3.4. Recognition Memory of the Stories (Questionnaire)

#### 3.4.1. Total Recognition Memory

An overall two-way ANOVA with group (SAD, control) and story type (AS, NS) as independent factors was first conducted to analyze the total recognition memory from the arousal and the neutral version of the story and then to analyze the total slide recognition in the different groups separately. 

The total recognition memory showed a main effect of story type on memory scores (F_1,116_ = 30.375, *p* < 0.0001; η^2^ = 0.209), indicating that participants recalled more slides from the AS compared with the NS. There was also a significant group effect on memory scores (F_1,116_ = 12.468, *p* < 0.001; η^2^ = 0.097), as the control group answered a total number of correct questions significantly higher than the SAD group (F_1,118_ = 9.890; *p* < 0.002). There was no group x story type interaction (F_1,116_ = 2.014, *p* = 0.159; η^2^ = 0.017).

**Figure 4:**
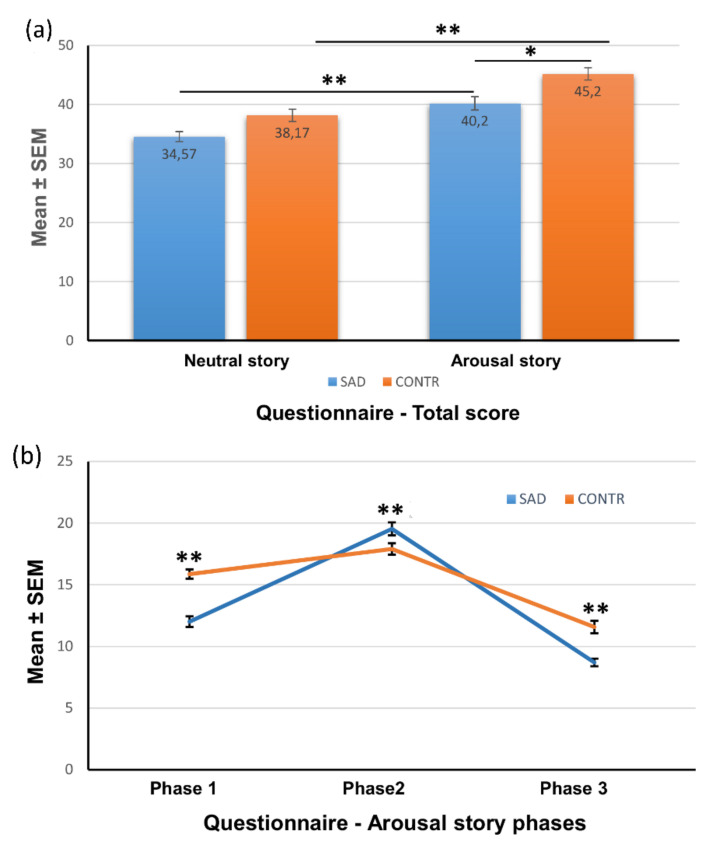
Recognition memory score. (**a**) Total score (mean ± SEM) of the questionnaire of the subjects assigned to the neutral and arousal conditions. * *p* < 0.01; ** *p* < 0.001. (**b**) Questionnaire scores (mean ± SEM) for each phase of the story of the subjects assigned to the arousal condition. * *p* < 0.05; ** *p* < 0.001.

We next explored the total recognition memory scores in the two groups separately. Both the SAD (F_1.58_ = 16.032; *p* < 0.001) and the control group (F_1,58_ = 109.350; *p* < 0.001) answered a significantly higher number of questions of the AS compared to the NS. The analyses of comparisons between the two groups revealed no significant differences for the NS (F_1,58_ = 3.404; *p* = 0.07, n.s.), although the SAD group answered fewer questions than the controls, while the control group reported a significantly higher number of correct answers of AS than the SAD group (F_1,58_ = 6.390; *p* < 0.01) (Figure 4a).

#### 3.4.2. Recognition Memory for Each Phase

We next examined the recognition memory in each phase of the stories using two-way ANOVA with group (SAD-AS, control-AS; SAD-NS, control-NS) as a between-subject factor and the story phase (1, 2, 3) as a within-subject factor. 

The overall ANOVA for all the three phases showed a main effect of story phase on memory scores (F_2,232_ = 86.084; *p* < 0.0001, η^2^ = 0.426) and a significant group x story phase interaction (F_6,232_ = 33.915; *p* < 0.001, η^2^ = 0.467).

A series of separate ANOVAs evidenced that the SAD group correctly answered a significantly smaller number of questions concerning neutral phases (1 and 3) than the control group (F_1,58_ = 10.417; *p* < 0.001 and F_1,58_ = 4.019 *p* < 0.05, respectively). In contrast, and according to the free-recall test, subjects with SAD answered a significantly higher number of questions related to the emotional phase (2) than the control group (F_1,58_ = 3.918; *p* < 0.05) (Figure 4b).

### 3.5. Correlational Analysis between GSS Values and Free-Recall Test and Recognition Test

Pearson’s correlation analysis was performed to analyze the correlation between the GSS scores and the free-recall and recognition memory scores. The analysis evidenced that in the SAD group, the GSS scores were negatively correlated both with the free-recall scores [r (60) = −0.806, *p* < 0.001] and the recognition memory scores [r (60) = −0.731, *p* < 0.001]. Therefore, a higher GSS score corresponds to worse memory performance. 

No correlation was found for the control group for free-recall [r (60) = −0.217, *p* = 0.096] or for the questionnaire [r (60) = −0.236, *p* = 0.070]. 

**Figure 5:**
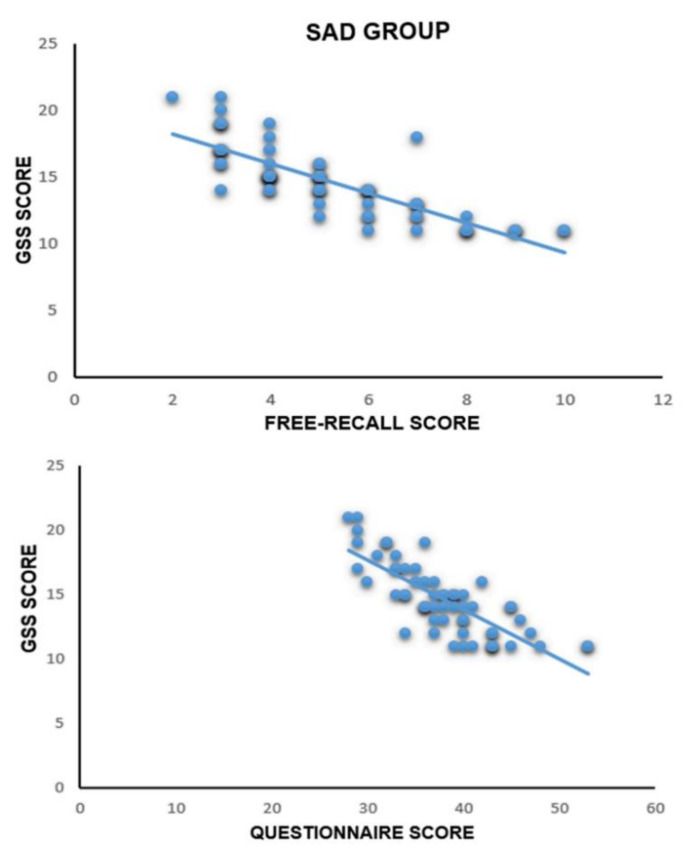
Correlation between GSS scores and free-recall and recognition memory scores in the SAD group. A negative relationship is shown both in the free-recall test and in the recognition memory test (questionnaire).

Significant results are shown in Figure 5, and the correlation analyses for all of the four groups are presented in Table 2.

## 4. Discussion

### 4.1. This Work

In this study, for the first time, state-dependent deficits in declarative memory are reported in a group of medically untreated women with SAD during the symptomatic phase compared with non-SAD controls. Our key findings suggest that an overall deficit exists in declarative memory performance and that subjects with SAD display selective activation of attentional processes toward negative, as opposed to neutral, stimuli. 

More accurately, our principal results showed that individuals with SAD (i) remembered a significantly smaller total number of details than the controls, both in the free-recall test and in the questionnaire, showing impaired memory processes, but (ii) they judged the AS as significantly more emotional than the control group, and (iii) they recalled a significantly higher number of slides and answered a significantly higher number of questions of the central phase of the AS compared to the controls. Moreover, (iv) a significant negative correlation was found in the SAD group between the GSS scores and the results for memory performance, with a higher GSS score corresponding to worse memory performance. Finally, (v) the SAD group showed a significantly higher score on the BDI-II than the control group, highlighting that, at least during the symptomatic period, SAD is a significant affective disorder. 

SAD influenced the reactivity to the emotional stimuli and, in fact, participants of the SAD-AS group showed a significantly higher memory performance in phase 2 of the AS compared to the control-AS group. However, the overall performance in the AS highlighted a significant impairment in the total memory score compared with the control-AS group. In the NS, although there was not a significant result in the total score, subjects of the SAD-NS group also reported a lower memory score compared with the control-NS group. Therefore, it is not surprising that we found a significant negative correlation between the GSS score and the memory score both in the overall SAD group and in the two groups separately, as SAD was present in both the SAD-AS and SAD-NS groups. Moreover, no correlation was found for the control group. 

Taken together, the data of this study support our hypotheses that SAD is characterized by impairment in declarative memory and attentional bias for emotional negative stimuli.

### 4.2. Contributions

Memory impairment is a frequent sign in nonseasonal depression [50,51]. In general, memory deficits in MDD range from mild, such as in cognitive speed, to very serious, dementia-like deficits in the more severe forms. In particular, memory for relevant information is reduced in MDD [26,27,52,53,54] and the impairment can be predictive of the functional outcome and correlated with the illness chronicity [55]. Patients with MDD exhibit decreased ability of retrieval in declarative memory [53,54,56], linked to the hippocampal functionality, and recent evidence suggests a dual role of the intermediate hippocampus in memory and affective information processes [57]. Moreover, MDD is characterized by a processing bias toward the negative aspect of the environment that can be considered a key component in depression [58].

The audio–visual story we used is a very useful tool for investigating both memory performance and reactivity to negative emotional stimuli in SAD. The normal behavior we expected in participants, in both the control and in the SAD group, was that they better remembered emotionally salient than neutral information, related to the central phase of AS. This is explained by the well-known relevance for the survival of the emotional salience of the stimuli. Taken together, the observed increase in remembering details of the central phase of AS and the impairment in the overall recall evidenced that SAD, similar to MDD, is characterized by negative attentional bias and by cognitive impairments. The attentional bias in SAD may play a role in the development and recurrence of SAD episodes, as if individuals show a higher memory encoding toward negative events, they should have a higher probability of experiencing a depressed mood. 

Our experimental plan differs from previous research because it focuses on long-term memory. In fact, to the best of our knowledge, the scientific literature lacks research on the effects of SAD on long-term memory, while few previous studies highlighted short-term memory impairments in SAD.

In particular, Sullivan and Payne (2007) [34] evidenced cognitive failures, using the Cognitive Failure Questionnaire, related to difficulties in everyday situations in controlled processes, such as attention and working memory. They analyzed the questionnaire results in subjects with SAD and MDD, finding statistically equivalent cognitive failures. Jensen et al., in a 2015 study [30] focusing on the development and validation of the Verbal Affective Memory Test-24, partly supported a working memory impairment in SAD. They found that individuals diagnosed with SAD showed a significant decrease in positive word recall during the symptomatic phase in the winter period compared to the summer period, while this was not the case for negative and neutral word recall.

Working memory impairments in SAD related to emotional information are also supported by the study of Hjordt et al. (2017) [28], who carried out the first longitudinal study with a seasonally counterbalanced design to distinguish stable cognitive traits from transient cognitive deficits during the symptomatic phase. The research showed that the deficits found in working memory, cognitive processing speed, and motor speed were characteristics present in SAD subjects regardless of the season. However, in this study, it was not possible to establish whether the observed cognitive deficits were present in the participants before the onset of the first depressive episode.

In another study, Hjordt et al. (2017) [29] found that individuals with SAD exhibited emotional bias toward negative affective stimuli during the symptomatic phase only.

Our data are comparable with the rate of SAD in the young population [59,60,61,62,63]. 

A recent study conducted by Lukmanji et al. (2020) [64] on a population of Canadian students found that young people may have an increased vulnerability to secondary depressive symptoms, potentially making them more susceptible to the development of SAD. In fact, symptoms can potentially interfere with the individual’s psychosocial functioning and development, impairing levels of concentration and self-esteem in young people, leading to significant impairment in school performance and potentially affecting academic abilities and social relationships.

Our results can be interpreted in the context of the dual vulnerability model first proposed by Young et al. (1991) [65] and then revisited by Lam et al. (2001) [66]. According to this model, SAD develops when an individual presents significant primary seasonal physiological symptoms (e.g., hypersomnia, hyperphagia, and low energy) along with a psychological vulnerability, leading the individual to be more susceptible to developing secondary cognitive-affective symptoms during the stress of the winter season. Therefore, the model considers SAD symptoms as a consequence of complex interactions between environmental changes, biological vulnerabilities, and psychological vulnerabilities (Lam et al., 2001; Young et al., 1991) [59,60]. 

The dual vulnerability hypothesis can help explain the discrepant results of the studies on SAD, taking into account the heterogeneity of the pathophysiology of this disorder, with a complex interplay of seasonal physiological symptoms and psychological vulnerability, which can vary among individuals with a history of SAD. In fact, SAD is likely to result from a vulnerability to experience physiological symptoms and from a psychological vulnerability to develop affective and cognitive symptoms.

### 4.3. Limitations

Our study presents, along with the strengths of new findings in the field of cognitive impairments in long-term memory in SAD, some limits that need to be considered in the interpretation of the results.

First, the study lacks repeated measurements within the year, so participants were tested only during the symptomatic period. The reason why it was not possible to repeat the experiment during the remission period in spring/summer is due to the specific experimental paradigm: the audio–visual stories we used, in particular the AS, cannot be administered twice to the same subject because the results of the free recall test and of the recognition memory test would be invalidated by having already viewed the story. 

A second limitation is that our study was limited to women, as they are much more likely than men to develop SAD, and this can lead to general conclusions with gender bias. In future studies, it will be important to investigate whether these findings are consistent across genders. It is well known that men and women can respond differently to emotional stimuli, and we cannot exclude that the bias in the encoding of emotional stimuli in SAD may depend on the female gender. Moreover, SAD has high comorbidity with premenstrual disorders [8], and we did not consider this factor.

Despite these limitations, we believe that our results provide a good basis from which to work.

### 4.4. Future Works

Cognitive impairments contribute to a worsening in the patient’s quality of life during the symptomatic period, so more research efforts are needed, in particular, to distinguish stable from transient characteristics of individuals with high levels of seasonality. To this aim, we are now conducting a long-term follow-up study, with repeated measurements of memory within the different seasons, using different emotional stimuli selected from the same standardized sets, such as the International Affective Picture, the Karolinska Directed Emotional Faces, and the Picture of Facial Affect of Ekman.

We will include both male and female subjects in future studies. 

## 5. Conclusions

In summary, the present study adds to the research by investigating the interplay of cognitive and emotional factors in SAD. The key findings suggest that subjects with SAD showed an impairment in declarative memory and an attentional bias toward negative stimuli, during the symptomatic period. Within our sample, composed of female university students, the severity of GSS score corresponds to worse memory score, highlighting that subjects with SAD are vulnerable to develop cognitive symptoms.

Four decades after the first descriptions of SAD, still many aspects remain to be clarified, especially concerning the distinction between stable and transient characteristics of individuals with SAD and, therefore, further development in this field will require longitudinal studies across different seasons.

The evidence of long-term memory impairment caused by the depressive states linked to SAD justify the continuation of studies, with the aim of better understanding this disorder and contributing to improve the quality of life of patients.

## Figures and Tables

**Table 1:** Demographic and clinical characteristics of the four groups. Data are presented as the mean ± SD. p = level of significance.

Group	Age (Years)	Years of Residence in Central/Southern Italy	GSS Score	BDI Score
Total (*n* = 120)	22.50 ± 2.676	18.19 ± 5.204	11.55 ± 4.470	7.44 ± 3.609
SAD-AS (*n* = 30)	22.20 ± 2.618	18.30 ± 6.193	14.37 ± 2.566	8.77 ± 3.431
SAD-NS (*n* = 30)	23.53 ± 3.298	17.77 ± 5.494	14.83 ± 2.902	8.83 ± 3.715
Contr-AS (*n* = 30)	21.90 ± 2.057	18.47 ± 3.037	8.67 ± 3.800	6.57 ± 3.390
Contr-NS (*n* = 30)	22.37 ± 2.414	18.23 ± 5.752	8.33 ± 3.726	5.60 ± 2.872
SAD-AS vs. Contr-AS	*p* = 0.647 (F_1,58_ = 0.244)	*p* = 0.895 (F_1,58_ = 0.018)	*p* < 0.001 (F_1,58_ = 46.366)	*p* < 0.01 (F_1,58_ = 6.241)
SAD-NS vs. Contr-NS	*p* = 0.123 (F_1,58_ = 2.440)	*p* = 0.749 (F_1,58_ = 0.103)	*p* < 0.001 (F_1,58_ = 56.827)	*p* < 0.001 (F_1,58_ = 14.137)

**Table 2:** Correlational analysis between GSS scores and free-recall and recognition memory scores for the four different groups.

Gruppo	r (Free-Recall)	r (Questionnaire)
SAD-NS (*n* = 30)	r (30) = −0.822, *p* ˂ 0.001	r (30) = −0.799, *p* ˂ 0.001
SAD-AS (*n* = 30)	r (30) = −0.844, *p* ˂ 0.001	r (30) = −0.743, *p* ˂ 0.001
Contr-NS (*n* = 30)	r (30) = −0.163, *p* = 0.438	r (30) = −0.355, *p* = 0.081
Contr-AS (*n* = 30)	r (30) = −0.231, *p* = 0.220	r (30) = −0.260, *p* = 0.165

r = Pearson’s correlation test.

## Data Availability

The data are available from the corresponding author upon reasonable request.

## Supplementary Materials

The following supporting information can be downloaded at: https://www.mdpi.com/article/10.3390/brainsci12101352/s1, Figure S1: Binary logistic regression predicting the likelihood of SAD diagnosis; Table S1: Estimate plots for the variables age, residence, GSS, and BDI.

## Abbreviations

**SAD**	Seasonal affective disorder
**MDD**	Major depressive disorder
**SPAQ**	Seasonal Pattern Assessment Questionnaire
**BDI-II**	Beck Depression Inventory-II
**NS**	Neutral story
**AS**	Arousal story
**GSS**	Global seasonality score
**ANOVA**	Analysis of variance
