# Supplementary material for: Declarative Memory Impairment and Emotional Bias in Recurrent Depression with a Seasonal Pattern: The Interplay between Emotion and Cognition in Seasonal Affective Disorder
Source: Brain Sci. 2022 Oct 5;12(10):1352. doi: 10.3390/brainsci12101352 (PMC9599318; doi:10.3390/brainsci12101352)
Supplement: Supplementary file 1 [file brainsci-12-01352-s001.zip › brainsci-1912917-supplementary.pdf]

Analysis of clinical and demographic characteristics of the sample with binary logistic regression

Binary logistic regression was used to examine whether age, residence, GSS, and BDI were associated with the likelihood to have a SAD diagnosis (Table 1).

A preliminary analysis suggests that the assumption of multicollinearity was met (tolerance = 0.97).

The model was statistically significant, χ^2 (4, N = 120) = 81.6, p < 0.001, (Table 1), suggesting that it could distinguish between cases with SAD and without SAD diagnosis.

The model obtained explained 49.0% (Cox & Snell R square) and 65.8 % (Nagelkerke R square) of the variance in the dependent variable and correctly classified 85% of cases.

No significant differences were evidenced between the SAD group and the controls concerning age (OR 1.10; 95%CI = 0.90, 1.33), and the number of years of residence in central and southern Italy (OR .1; 95%CI = 0.90, 1.10). Significant differences were showed for BDI (OR 1.21; 95%CI = 1.03, 1.43; p < 0.02) and GSS (OR 1.81; 95%CI = 1.44, 2.28; p < 0.001).

As shown in table 1, GSS and BDI variables, but not age and residence, contributed to the model.

The GSS odds ratio suggests that, for every increase in GSS, participants were more likely to have a SAD diagnosis, as well as for every increase in BDI.

The estimate plots are shown in figure 1.

Therefore, the analysis with binary logistic regression supports the results presented in the manuscript.

Table S1. Binary logistic regression predicting the likelihood of SAD diagnosis. Estimates represent the log odds of Group SAD vs Group of control.

Model Fit Measures

					Overall Model Test		
Model	Deviance	AIC	BIC	R^2_{McF}	χ^2	df	p
1	84.8	94.8	109	0.490	81.6	4	< .001

Model Coefficients - Group

						95% Confidence Interval	
Predictor	Estimate	SE	Z	p	Odds ratio	Lower	Upper
Intercept	−10.82896	3.1833	−3.4018	< .001	1.98e−5	3.87e−8	0.0102
Age	0.09841	0.0987	0.9965	0.319	1.10	0.909	1.3390
Residence	0.00370	0.0503	0.0735	0.941	1.00	0.909	1.1077
BDI	0.19249	0.0836	2.3022	0.021	1.21	1.029	1.4281
GSS	0.59587	0.1171	5.0883	< .001	1.81	1.442	2.2828

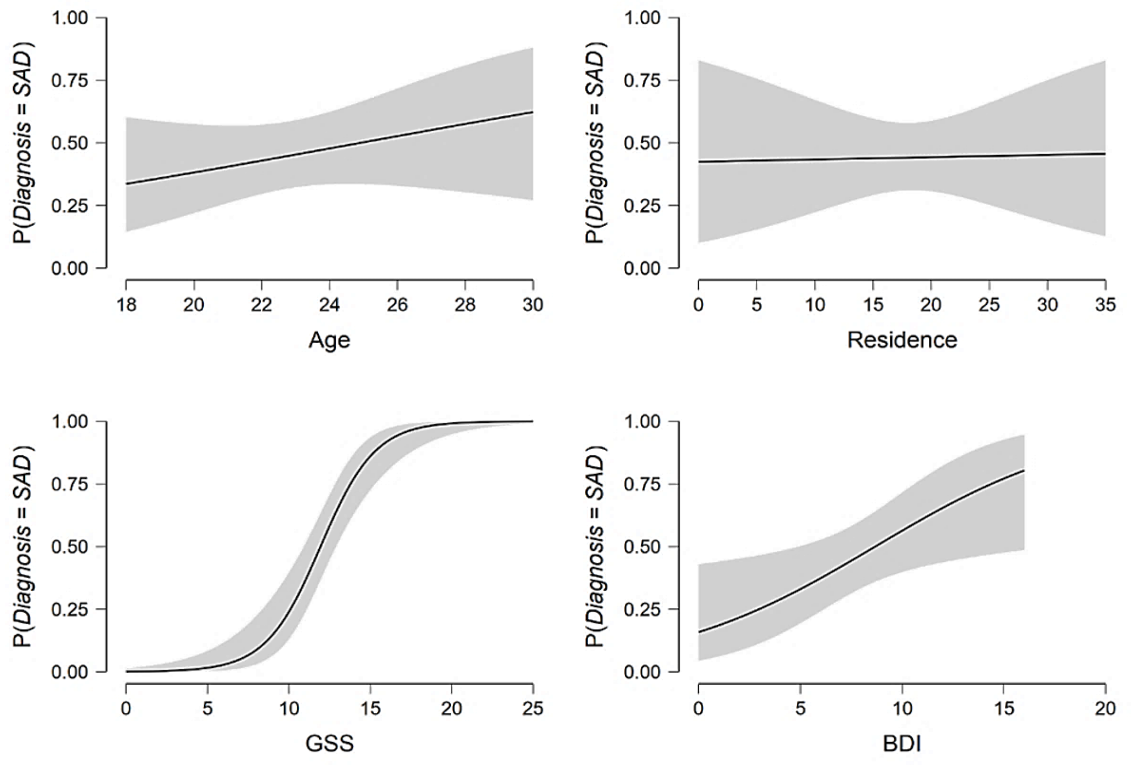

Figure S1. Estimate plots for the variables age, residence, GSS, and BDI
